# Detection of bornavirus-reactive antibodies and BoDV-1 RNA only in encephalitis patients from virus endemic areas: a comparative serological and molecular sensitivity, specificity, predictive value, and disease duration correlation study

**DOI:** 10.1007/s15010-023-02048-1

**Published:** 2023-05-30

**Authors:** Petra Allartz, Sven-Kevin Hotop, Birgit Muntau, Alexander Schlaphof, Corinna Thomé-Bolduan, Martin Gabriel, Nadine Petersen, Maren Lintzel, Christoph Behrens, Petra Eggert, Kirsten Pörtner, Johann Steiner, Mark Brönstrup, Dennis Tappe

**Affiliations:** 1 Bernhard Nocht Institute for Tropical Medicine, Bernhard-Nocht-Str. 74, 20359 Hamburg, Germany; 2 Helmholtz Centre for Infection Research, Braunschweig, Germany; 3 Department of Infectious Disease Epidemiology, Robert Koch Institute, Berlin, Germany; 4 Department of Psychiatry and Psychotherapy, University Hospital Magdeburg, Magdeburg, Germany; 5 German Center for Infection Research (DZIF), Site Hannover-Braunschweig, Braunschweig, Germany

**Keywords:** Bornavirus, Serology, PCR, IgG1, Peptide

## Abstract

**Purpose:**

Human Borna disease virus (BoDV-1) encephalitis is an emerging disease in Germany. This study investigates the spectrum of human BoDV-1 infection, characterizes anti-BoDV-1-antibodies and kinetics, and compares laboratory test performances.

**Methods:**

Three hundred four encephalitis cases, 308 nation-wide neuropsychiatric conditions, 127 well-defined psychiatric cases from Borna disease-endemic areas, and 20 persons with contact to BoDV-1 encephalitis patients or animals were tested for BoDV-1 infections by serology and PCR.

**Results:**

BoDV-1 infections were only found in encephalitis patients with residence in, or recent travel to, virus-endemic areas. Antibodies were detected as early as 12 days after symptom onset. Serum antibody levels correlated with disease duration. Serology was ordered after 50% of the disease duration had elapsed, reflecting low awareness. BoDV-1-antibodies were of IgG1 subclass, and the epitope on BoDV-1 antigens was determined. Specificity of the indirect immunofluorescence antibody test (IFAT) and lineblot (LB) from serum and cerebrospinal fluid (CSF), as well as PCR testing from CSF, was 100%. Sensitivity, depending on first or all samples, reached 75–86% in serum and 92–94% in CSF for the IFAT, and 33–57% in serum and 18–24% in CSF for the LB. Sensitivity for PCR in CSF was 25–67%. Positive predictive values were 100% each, while negative predictive values were 99% (IFAT), 91–97% (LB), and 90% (PCR).

**Conclusions:**

There is no hint that BoDV-1 causes other diseases than encephalitis in humans. Awareness has to be increased in virus-endemic areas. Tests are robust but lack sensitivity. Detection of IgG1 against specific peptides may facilitate diagnosis. Screening of healthy individuals is likely not beneficial.

## Introduction

The Borna disease virus 1 (BoDV-1) is a zoonotic virus of the *Bornaviridae* family that is long known for causing Borna disease (BD) in animals, an often fatal meningomyeloencephalitis of mainly horses and sheep. In 2018, BoDV-1 was shown to cause severe and mostly fatal encephalitis in humans in Germany [1, 2]. The virus is harbored at least by the bicolored white-toothed shrew (*Crocidura leucodon*) as a natural reservoir and infected shrews excrete the virus through feces, urine, saliva or skin [3–5]. The direct detection of zoonotic bornaviruses became notifiable in Germany in 2020 and as of early 2023, nearly 50 human BoDV-1 encephalitis cases are registered. Most cases were detected in retrospective studies. In 2021, the so far yearly maximum of seven acute cases were reported to public health authorities, but the disease still appears to be under-recognized due to lack of awareness. The region endemic for BoDV-1 in Germany extends from Bavaria in the south to further northern and eastern federal states [6]. Decades ago, a controversy about the involvement of BoDV-1 in psychiatric diseases emerged, but dedicated blinded case–control studies—however outside the BD endemic area—and a meta-analysis among other publications could not confirm a role of BoDV-1 in such diseases [7–9].

Here, we tested various patient cohorts for BoDV-1 infections, encompassing encephalitis cases and various neuropsychiatric conditions nation-wide, well-defined psychiatric cases from BD endemic areas, as well as persons with contact to BoDV-1 diseased patients and animals. We conducted comparative laboratory test performance analyses including calculation of specificity and sensitivity, and positive and negative predictive values (PPV, NPV), for an indirect immunofluorescence antibody test (IFAT) and lineblot (LB) from serum and CSF, as well as for a quantitative real-time reverse transcription polymerase chain reaction assay (qPCR) from CSF. In addition, IgG subclass testing and antibody epitope mapping for BoDV-1 N and P antigens were performed in positive cases from serum and/or CSF. Antibody levels and PCR results were correlated to the length of disease, and the time of antibody presence is demonstrated.

## Materials and methods

### Cohorts, materials, workflow, statistics, and ethical clearance

The encephalitis patient cohort encompassed 12 known and molecularly confirmed BoDV-1 cases (25 sera and 15 CSF samples) and 2 known probable BoDV-1 cases (16 sera and 16 CSF samples), diagnosed according to the proposed case definition [10] at the Bernhard Nocht Institute between 2018 and 2022. Patient age was 62 years in median (range 6–79) at symptom onset, 8 were male and 6 female. Additionally, 290 patients with encephalitis of unknown origin from all over Germany (236 sera and 227 CSF samples) were included. Further cohorts consisted of 308 individuals with various neuropsychiatric conditions from all over Germany (271 sera and 37 CSF samples; chronic fatigue syndrome, vigilance decline, cognitive dysfunction, personality change, epilepsy, vertigo, and neuropathy), 127 patients with well-defined psychiatric disorders from a region endemic for animal BD in the East of Germany (127 sera and 80 CSF samples; from patients with schizophrenia, depression, and bipolar disorder), 10 household members of known BoDV-1 encephalitis cases, and 10 healthy persons with contact to known BoDV-1 infected animals (horses and alpacas) in endemic regions.

All serum and CSF samples underwent testing for bornavirus-reactive antibodies by IFAT and LB following a diagnostic testing scheme for the rapid intra vitam diagnosis of human bornavirus encephalitis, in parallel with a graded case definition for possible, probable and confirmed BoDV-1 encephalitis [10]. Sera from 200 healthy blood donors, 16 patients with possibly test-interfering (auto-)antibodies (EBV and CMV infection, malaria, rheumatoid factor (RF) or antinuclear antibody (ANA) positive) were tested in addition. All CSF samples underwent analysis for BoDV-1 RNA by qPCR. IFAT positive serum samples were subjected to IgG subclass testing and IFAT positive serum and CSF samples underwent antibody epitope mapping for BoDV-1 N and P antigens. In patients with a positive serology or PCR test, results were correlated with the individual time point of illness (Spearman rank test). Specificity was calculated by dividing correct negatives by correct negatives + false positives, sensitivity was calculated by dividing correct positives by correct positives + false negatives, the positive predictive value was computed by dividing correct positives by correct positives + false positives, and the negative predictive value was computed by dividing correct negatives by correct negatives + false negatives; each per assay and type of material. Statistical analyses were performed with GraphPad Prism (version 9, GraphPad Software Inc., San Diego, CA). Details of statistical tests are provided in the respective figure legends. Laboratory testing was performed in the framework of routine diagnostics; ethical clearance for data analysis of positive cases was granted from the Medical Board of Hamburg (No. PV5616).

### Indirect immunofluorescence IgG antibody test

For the IFAT, Crandell-Rees Feline Kidney (CRFK) cells persistently infected with BoDV-1 strain V and uninfected cells of the same cell line as controls were used [10]. Patient IgG antibodies were detected using an anti-human IgG conjugate (Euroimmun, Lübeck, Germany, undiluted as per manufacturer’s instructions). All sera or CSF samples with intranuclear IFAT patterns indicative for bornavirus infections [11] in dilutions ≥ 1:10 were considered positive. Sera and CSF samples of patients with confirmed BoDV-1 encephalitis served as positive controls, whereas a pooled serum of 20 healthy blood donors was used as negative control. Slides were read by at least two experienced staff.

### IgG lineblot

The LB employs recombinant phosphoprotein (P) and nucleoprotein (N) antigen of both BoDV-1 and VSBV-1 with an adjusted cut-off of 12 arbitrary units (AU) each [10–12] as validated by the technical manufacturer (Euroimmun, Lübeck, Germany) using bornavirus-positive sera and CSF samples in comparison to > 200 controls without evidence of bornavirus encephalitis. Patient IgG antibodies were detected using an anti-human IgG conjugate (Euroimmun). The LB was considered positive when both antibodies against N and P of BoDV-1 were positive, as well as cross-reactive antibodies against VSBV-1 N and P. Sera and CSF samples of patients with confirmed BoDV-1 encephalitis served as positive controls, whereas a pooled serum of 20 healthy blood donors was used as negative control. AU of patient IgG were automatically calculated using the EUROLineScan software (Euroimmun).

### Immunoglobulin G subclass testing

IgG subclass testing was performed by IFAT using IgG IFAT positive sera from patients with BoDV-1 encephalitis. Therefore, Fc subclass-specific mouse-anti human IgG1, IgG2, IgG3 and IgG4 (Nordic-MUbio, Susteren, The Netherlands) were employed in 1:50 dilutions. Due to much lower amounts of CSF available, this method was not employed to CSF.

### Antibody epitope mapping for BoDV-1 N and P antigens

IgG antibodies from sera and CSF of the BoDV-1 encephalitis patients, as well as from sera of 20 healthy blood donors, were protein G-purified using affinity chromatography. Purified antibodies were visualized on a reducing SDS gel and subjected to peptide microarrays at a concentration of 5 µg/ml, similar to a procedure performed for herpes B virus and VSBV-1 previously [12, 13]. 543 15-mer peptides were synthesized spanning the whole P and N antigen sequence of BoDV-1 (GenBank accession QWC48963 and QWC48965) with an offset of one amino acid (AA) using the synthetic peptide arrays on membrane support technique [14] and printed onto glass slides with the SC2 method [15]. After incubation with samples and secondary antibodies conjugated to a fluorescent dye, slides were scanned with a DNA microarray scanner (Agilent Technologies, Waldbronn, Germany). The resulting pictures were obtained using the Agilent Feature Extraction Software version 12.2.0.7, and Chimera [16] for 3D illustrations.

### RNA extraction and BoDV-1 polymerase chain reaction testing

RNA from CSF was extracted using the QiaAmp Viral RNA MiniKit (Qiagen, Hilden, Germany). Molecular testing was performed by quantitative reverse-transcription real time polymerase chain reaction (qRT-PCR) for BoDV-1 [17] from CSF. Cycle threshold (ct) values were calculated as the second derivative of the melting curve. BoDV-1 copy numbers were calculated with the volume of the RNA extract used for the PCR reaction and the standard curve of the titrated synthetic positive control oligonucleotide (GACTCCCTGGAGGACGAAGAAGATGACCCAGACACTACGACGGGAACGATCAGGTCACCAAGACCACGGAAGTCA) with known molarity and copy numbers. The limit of detection was calculated by titrating the positive control.

## Results

### Bornavirus-reactive antibodies and viral RNA in patient cohorts

Bornavirus-reactive antibodies in the IFAT, or in the IFAT and the LB were only found in the known patient cohort with confirmed or probable bornavirus encephalitis with residence in, or recent travel to, virus-endemic areas in Germany (14 cases of a total of 304 encephalitis cases, 4.6%). BoDV-1 RNA was only detected in CSF of the patient group with BoDV-1 encephalitis.

No bornavirus-reactive antibodies were detected by either assay among the patient cohort with various neuropsychiatric conditions from all over Germany, and also not in individuals with specific neuropsychiatric disorders from a virus-endemic region in the East of Germany. No bornavirus-reactive antibodies were found in healthy household members of known BoDV-1 encephalitis cases and healthy persons with contact to known BoDV-1 infected animals in endemic regions either. Healthy blood donors and patients with possibly test-interfering (auto-)antibodies did not show serological reactions to bornavirus antigens by the assays.

**Fig. 1:**
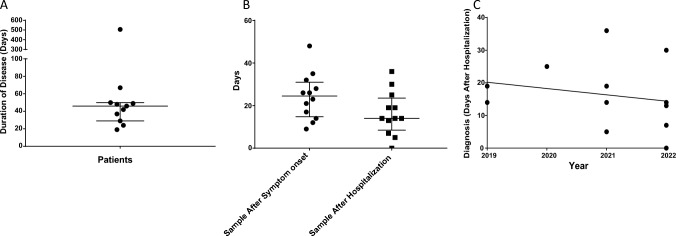
Disease duration in confirmed BoDV-1 encephalitis cases, time point of taking diagnostic samples, and time point of diagnosis over years. **A** Duration of BoDV-1 encephalitis in the patient cohort. Median length of illness was 46 days. The two patients with the longest disease duration were severely immunosuppressed as therapeutic approach for BoDV-1 encephalitis. Median with interquartile range is shown. **B** Time point of taking diagnostic samples. Median duration before a sample was taken after symptom onset was 25 days, corresponding to median day 14 after hospital admission. Median with interquartile range is shown. **C** Diagnosis at days after hospitalization. A weak tendency to request bornavirus serology earlier each year is seen over time (linear regression; R^2^=0.045)

Duration of BoDV-1 encephalitis was in median 46 days (range 19–506 days; one patient is still alive and excluded from the analysis). The first serological sample tested for a BoDV-1 etiology was requested and analyzed 25 days in median after symptom onset (range 9–48 days), and in median 14 days after hospitalization (range 0–36 days; Fig. 1A and B). One patient was serologically diagnosed on the day of his death. During the ongoing awareness campaign in Germany for BoDV-1 encephalitis, a tendency to consider BoDV-1 as etiology in encephalitis of unknown origin after hospitalization is seen earlier every year (Fig. 1C).

### Presence and height of antibody levels and correlation of serology with duration of disease

**Fig. 2:**
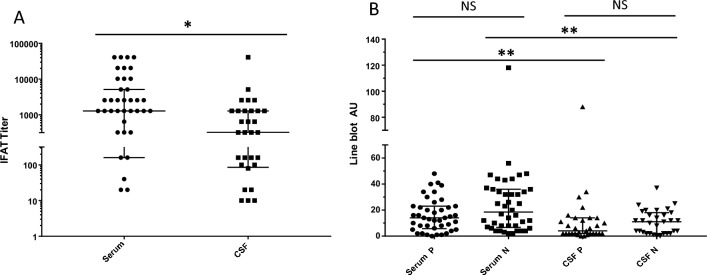
Height of antibody levels in BoDV-1 patients. **A** Immunofluorescence antibody test (IFAT) results for serum and cerebrospinal fluid (CSF) of all BoDV-1 patients tested in this study. Serum titers are significantly higher than CSF titers (*p < 0.05; Mann–Whitney test). Median with interquartile range is shown. **B** Line blot antibody test results for serum and CSF of all BoDV-1 patients. Serum antibodies against individual antigens are significantly higher than respective CSF antibody levels (**p < 0.005; Mann–Whitney test (respective one-on-one comparison)). Median with interquartile range is shown. *AU* arbitrary units; *NS* not significant

**Fig. 3:**
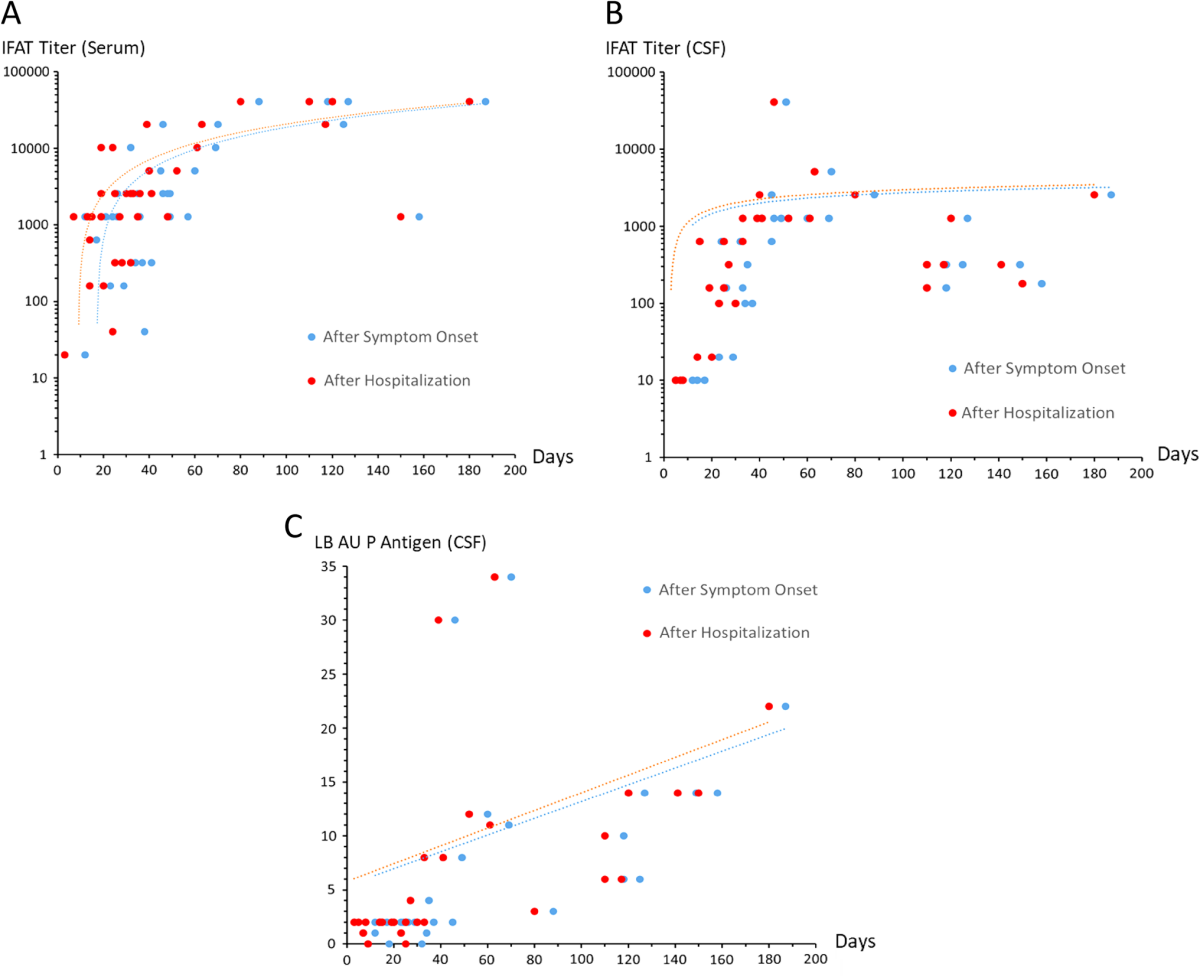
Height of antibody levels in relation to disease duration. **A** Immunofluorescence antibody test (IFAT) results for serum of all BoDV-1 patients tested in this study over time. Correlation of titers with time after symptom onset is 0.67 and after hospitalization 0.72. Logarithmic scale. **B** IFAT results for cerebrospinal fluid (CSF) of all BoDV-1 patients tested in this study over time. Correlation of titers with time after symptom onset is 0.62 and after hospitalization 0.63. Logarithmic scale. **C** Line blot (LB) arbitrary units (AU) of antibodies against BoDV-1 P antigen in CSF of all BoDV-1 patients tested in this study. Correlation of AU with time after symptom onset and after hospitalization is 0.78 each. Linear scale

Antibody titers in the IFAT and AUs in the LB were significantly higher in serum than in CSF (*p* < 0.05; Fig. 2A and B); no significant difference was seen for N versus P AUs in the same sample material (Fig. 2B). IFAT titers and AUs of antibodies against N and P in serum and CSF increased during the course of disease. Antibody levels correlated with the individual length of disease at the time point when the sample was taken (rho value for day after symptom onset and day after hospitalization (Spearman rank test), respectively): serum IFAT titers 0.67 and 0.72, CSF IFAT titers 0.62 and 0.63, serum AUs (N) 0.44 and 0.48, serum AUs (P) 0.58 and 0.59, as well as CSF AUs (N) 0.62 and 0.64, and CSF AUs (P) 0.78 and 0.78 (Fig. 3A–C with data for the IFAT in serum and CSF and AU of anti-P in CSF).

**Fig. 4:**
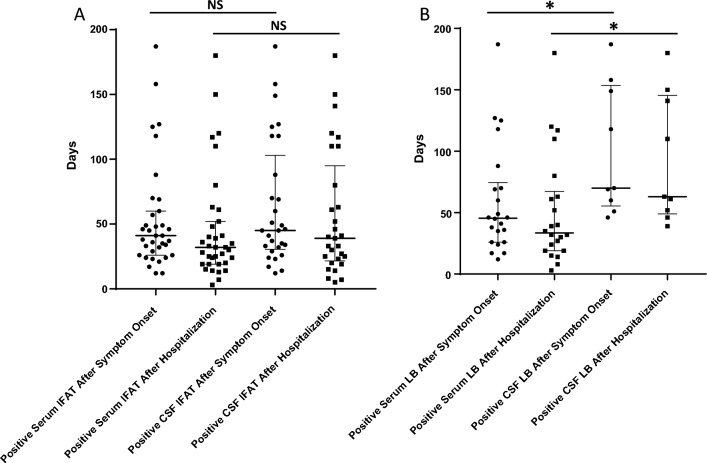
Presence of antibodies at time points of disease. **A** Positive immunofluorescence antibody test (IFAT) result for serum and cerebrospinal fluid (CSF) of all BoDV-1 patients tested in this study at given time points in days after symptom onset and hospital admission. Antibodies in serum were non-significantly earlier positive than in CSF. Median with interquartile range is shown. **B** Positive line blot (LB) results for serum and CSF of all BoDV-1 patients tested in this study at given time points in days after symptom onset and hospital admission. Antibodies in serum were significantly earlier positive than in CSF (*p < 0.05). Median with interquartile range is shown

Antibodies were detected by IFAT in serum in median on day 41 after symptom onset and day 32 after hospitalization (12–187 and 3–180), and in CSF in median on day 45 after symptom onset and day 39 after hospitalization (12–187 and 5–180; Fig. 4A). A positive LB result was shown in serum in median on day 45 after symptom onset and day 35 after hospitalization (12–187 and 3–180), and in CSF in median on day 65 after symptom onset and day 60 after hospitalization (46–187 and 39–180; Fig. 4B). A real seroconversion was observed in follow-up serum samples of three patients (days 12, 24, 38 after symptom onset and days 3, 15, 24 after hospitalization) in the IFAT. In general, antibodies measured by either method appeared earlier in serum than in CSF (not significant for the IFAT (*p* = 0.47 and 0.4), but significant for the LB (*p* = 0.01 and 0.01)). In serum, antibodies against N were earlier positive than antibodies against P in six samples (four samples the other way round), while in CSF, antibodies against N were earlier positive than antibodies against P in seven samples (one sample vice versa). In the majority of samples, however, antibodies against N and P (and therefore considered a positive test outcome), were simultaneously present.

### Immunoglobulin G subclass testing

Testing of IFAT positive sera showed reactivity with anti-IgG1 conjugate in the IFAT in all patients. In contrast, testing with anti-IgG2, IgG3 and IgG4 conjugates was negative in all samples tested.

### Antibody epitope mapping for BoDV-1 N and P antigens

**Fig. 5:**
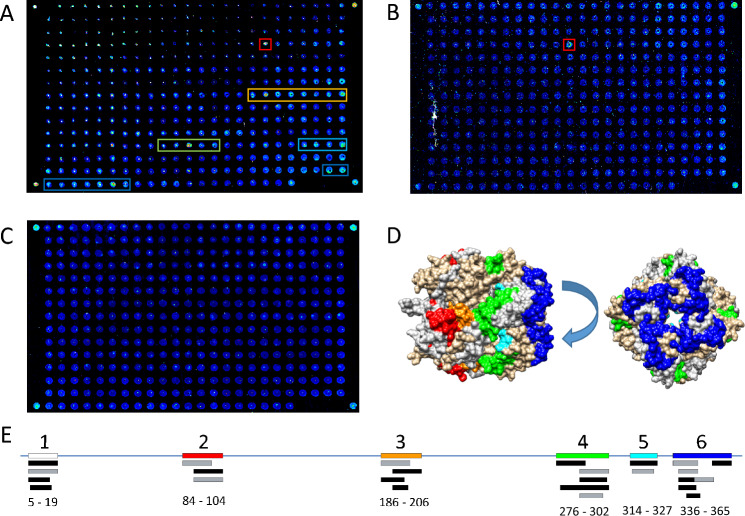
BoDV-1 N epitope mapping. **A** Serological fingerprint of a serum sample on the peptide microarray, represented as a 16-bit pseudocolor image obtained from the 650 nm channel. The detected antibody targets are designated by colored boxes if they were detected in two independent array experiments. **B** CSF serological fingerprint from same patient as in A showed a strong single spot bound antibody, binding to the same region as in A (red box). **C** Serum sample without observable signals. **D** Mapping of antibody target sequences onto the structure of the N protein (1N93). The colors correspond to peptide binding regions as found in A. **E** Overlay of antibody target sequences on the N protein revealing six immunogenic sites. The amino acid numbers are given below. Colors of the regions correspond to those in A and D. Region 1 is not resolved in the crystal structure (white box). Black boxes designate antibodies found in serum, gray boxes indicate antibodies from CSF samples

**Fig. 6:**
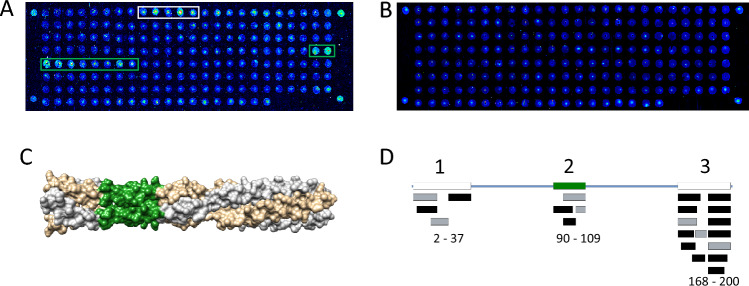
BoDV-1 P epitope mapping. **A** Serological fingerprint of a CSF sample on the peptide microarray, represented as a 16-bit pseudocolor image obtained from the 650 nm channel. **B** Serum sample without detectable signals. **C** Mapping of antibody target sequences onto the structure of the P protein (8B8A), color correspond to AA sequence found in A. **D** Overlay of antibody target sequences on the P protein revealing three immunogenic sites. The amino acid numbers are given below. The colors of the regions correspond to those in A and C. Regions 1 and 3 are not resolved in the crystal structure (white boxes). Black boxes represent antibodies found in serum, gray boxes indicate antibodies from CSF samples

From the total sample set, 12 different samples from 8 patients were examined via peptide microarrays. Mapping of antibody target sequences on the nucleocapsid N and phosphoprotein P antigens showed antibodies against both antigens. In total, nine antigenic sites on both proteins were recorded. Six sites were on the N protein (Fig. 5), whereas on the P protein, three immuno-dominant regions on both N- and C-terminal regions as well as a single region in the middle of the sequence could be observed (Fig. 6). The distribution of antibodies on the antigens showed higher prevalence for the P protein, especially when compared to size: 22 occurrences on 370 AA of N vs. 25 occurrences on the smaller, 201 AA long P protein imply that P was more immunogenic. Overall, most detected antibodies were derived from serum (68% of the antibodies against the N protein and 60% of those against the P protein), while the remaining were from CSF. All regions were located on the surface of the N (1N93) and P (8B8A) proteins. However, it should be noted that not all regions were resolved in crystal structures [18, 19]. The mapping revealed that in 9 out of 12 (75%) samples, antibodies against a linear sequence on the C-terminal end of the phosphoprotein were present. Remarkably, antibodies targeting different sequences could also be observed in serum and CSF in samples of the same patients (Fig. 5A and B; Table 1). Overall, in 2 out of 12 samples it was not possible to detect signals (titers 1:2560 and 1:160). We therefore conclude that these samples do not contain antibodies against linear epitopes and need intact 3D conformation of the proteins to bind. Spots on partial linear epitopes were however also detected by some of the control sera.

**Table 1:** Amino acid sequences of epitopes recognized on BoDV-1 P and N proteins by patient antibodies

	BoDV-1 P protein epitopes			BoDV-1 N protein epitopes		
Sample	Start AA	Sequence	End AA	Start AA	Sequence	End AA
1	24	ERSGSPRPRKIPRN	37	5	RRLVDDADAME	15
	168	VGTSAPMLPSHPAP	181	192	EAIDWINGQPWVGSF	206
	187	QLPSAPTADEWDII	200	289	PDAIKLAPRSFPNL	302
		No result		314	NPTMAGYRASTIQP	327
2	187	QLPSAPTADEWDII	200	5	RRLVDDADAMEDQDL	19
3	90	AEEVRGTLGDIS	101	5	RRLVDDADAMEDQDL	19
	168	VGTSAPMLPSHP	179	339	YRRREISR	346
	187	QLPSAPTADE	196	356	EISAIMKMIG	365
4	96	TLGDISARIEAGFE	109	5	RRLVDDADAMEDQDL	19
	168	VGTSAPMLPSHP	179	90	FVHGGVPRESYLSTP	104
	187	QLPSAPTADEWDII	200	336	LARYRRREISRGE	348
5		No result			No result	
6	96	TLGDISAR	103	90	FVHGGVPRESYLSTP	104
	170	TSAPMLPSH	178	186	QMFNPHEAIDWINGQ	200
	187	QLPSAPTADEWDII	200	276	HADLFPFLGAIRHPD	290
		No result		288	HPDAIKLAPRSFPNL	302
		No result		315	PTMAGYRASTI	325
		No result		336	YRRREISRGE	348
		No result		347	GEDGAELSGE	356
7	4	RPSSLVDSLEDEE	16	6	RLVDDADAMED	16
	187	QLPSAPTADEWDII	200		No result	
8	13	EDEEDPQTLRR	23		No result	
	104	IEAGFE	109		No result	
9		No result			No result	
10	168	VGTSAPMLPS	177	186	QMFNPHEAIDWI	197
	187	QLPSAPTADE	196	276	HADLFPFLGAIRHPD	290
		No result		288	HPDAIKLAPRSFPN	301
		No result		339	YRRREISRG	347
11	177	SHPAPPRI	184	90	FVHGGVPRESYLSTP	104
	187	QLPSAPTADEWD	198	192	EAIDWING	199
		No result		278	DLFPFLGAIRH	288
		No result		288	HPDAIKLAPRSF	299
		No result		343	EISRGED	349
12	2	ATRPSSLVDSLEDEE	16	84	PGLHAAFVHGGVPRE	98
	179	PAPPRIY	185		No result	

*AA* amino acid

### Laboratory test performances

#### Indirect immunofluorescence antibody test

##### Testing of serum

Specificity was 100% in the hands of well-trained staff. The sensitivity in the first sample of a BoDV-1 case was 75%, while in all samples together it was 86%. PPV was 100%, whereas NPV was 99%.

##### Testing of cerebrospinal fluid

Specificity was also 100% in the hands of well-trained staff. The sensitivity in the first sample of a BoDV-1 case was 92%, while in all samples together it was 94%, and thus higher than in serum, biased by the higher availability of serum samples at an earlier time point than CSF. PPV was 100%, and NPV was 99%. See Table 2 for summary.

**Table 2:** Characteristics of assays employed

		Specificity		Sensitivity		Pos. prediction		Neg. prediction	
Test	Samples*	Serum	CSF	Serum	CSF	Serum	CSF	Serum	CSF
IFAT	Initial sample	100%	100%	75%	92%	n.d	n.d	n.d	n.d
	All samples	100%	100%	86%	94%	100%	100%	99%	99%
LB	Initial sample	100%	100%	33%	18%	n.d	n.d	n.d	n.d
	All samples	100%	100%	51%	23%	100%	100%	97%	91%
	IFAT positives	100%	100%	57%	24%	n.d	n.d	n.d	n.d
qPCR	Initial sample	n.d	100%	n.d	55%	n.d	n.d	n.d	n.d
	All samples	n.d	100%	n.d	39%	n.d	100%	n.d	90%
	IFAT positives	n.d	100%	n.d	25%	n.d	n.d	n.d	n.d
	IFAT negatives	n.d	100%	n.d	67%	n.d	n.d	n.d	n.d

*Initial sample: only the first sample of a confirmed or probable BoDV-1 encephalitis case

*All samples: all samples from all cohorts

#### Lineblot

#### Testing of serum

Specificity was 100% when the above described N + P scheme was applied. The sensitivity in the first sample of a BoDV-1 case was 33%, while in all samples together it was 51%. When tested after a positive IFAT result, sensitivity was 57%. PPV was 100%, whereas NPV was 97%.

#### Testing of cerebrospinal fluid

Specificity was also 100% when the above described N + P scheme was applied. The sensitivity in the first sample of a BoDV-1 case was 18%, while in all samples together it was 23%. When tested after a positive IFAT result, sensitivity was 24%. PPV was 100%, whereas NPV was 91%. See Table 2 for summary.

#### Quantitative real-time reverse transcription polymerase chain reaction testing

#### Testing of cerebrospinal fluid

**Fig. 7:**
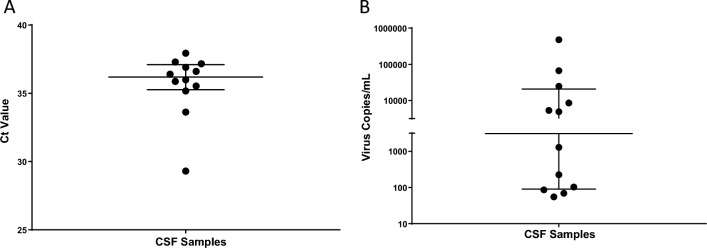
Distribution of BoDV-1 qPCR cycle threshold (ct) values and viral copy numbers in cerebrospinal fluid. **A** ct values with 36.2 in median, ranging from 29.3 to 37.9 are displayed. The two samples with the lowest ct values were from patients with the second shortest duration of disease when the material was taken and with the lowest antibody levels in CSF. Median with interquartile range is shown. **B** BoDV-1 copy numbers with 3086/mL in median, ranging from 55 to 477,844/mL are demonstrated. Median with interquartile range is shown

Specificity was 100%. The sensitivity in the first sample of a BoDV-1 case was 55%, while in all samples together it was 39%. Sensitivity in IFAT-positive samples was 25%, in contrast to 67% in IFAT-negative samples. PPV was 100%, whereas NPV was 90%. See Table 2 for summary. The limit of detection was 3.4 copies/reaction. Ct values from patient CSF samples were 36.2 in median and ranged from 29.3 to 37.9 (Fig. 7A). Copy numbers were 3086/mL in median, and ranged from 55 to 477,844/mL (Fig. 7B). There was no good correlation between ct values or copy numbers and day after symptom onset or day after hospitalization for all and initial PCR positive CSF samples (Spearman rank test, rho = 0.25–0.54; higher correlation for initial samples). There was no good correlation for CSF IFAT titer height and ct values or copy numbers either (Spearman rank test, rho = 0.32).

## Discussion

In our study of testing patient cohorts for bornavirus infections, paralleled by laboratory test performance analyses, we show that bornavirus-reactive antibodies were only detected in patients with encephalitis and with residence in, or recent travel to, known BoDV-1 endemic areas in Germany, corroborating recent more limited findings [10]. In only 4.6% of all cases with encephalitisa bornavirus etiology was shown in our study, which is, however, not representative as no systematic sampling was conducted. The demonstration of bornavirus-reactive antibodies only in encephalitis patients and thus highly symptomatic individuals once more reflects the high clinical manifestation index of BoDV-1 encephalitis in humans, as reported previously [11]. This renders seroprevalence studies among healthy individuals unimportant, an assumption which is further underscored by the negative findings in contact household members shown here and elsewhere [20, 21], as well as in healthy persons with contact to BoDV-1 infected animals described here and in a previous report [22], and after healthcare-associated exposure [23].

In addition, our study confirms the recently described absence of bornavirus-reactive antibodies in patients with neurological or neuropsychiatric conditions other than encephalitis [10], with much larger respective patient cohorts. Of note, we here specifically also tested psychiatric patients from an area endemic for BoDV-1 in the East of Germany. Absence of such antibodies in psychiatric patients was described years before [7, 8] in times of scientific dispute about the controversial involvement of BoDV-1 in psychiatric disease [9, 24], but testing had not been performed in an endemic area. Our negative findings do not rule out that BoDV-1 causes neuropsychiatric symptoms during encephalitis however; in the initial phase of BoDV-1 encephalitis, hallucinations and behavior changes are well noted [25–27].

Thus, positive serological findings in patients without encephalitis or in patients outside the known endemic area should be treated cautiously. Such test results are suspicious for being unspecific and patients should be further examined (re-testing and taking careful travel history). A BoDV-1 encephalitis case outside the known endemic area with a positive and specific serology from the West of Germany lately [26] illustrates this already anticipated scenario [6, 10]. The patient had a brief travel history to a rural endemic area well within the anticipated incubation time of several weeks to months (unpublished results).

We here show in our BoDV-1 encephalitis cohort a median disease duration of 46 days. It took 25 days in median after symptom onset (14 days after hospital admission) to consider a BoDV-1 etiology for the patient’s encephalitis, reflecting a 50% loss of time for possible treatment attempts. Moreover, as an example with so far limited data, the efficacy of ongoing awareness campaigns for BoDV-1 encephalitis tailored for physicians [28] was measured. A tendency to request bornavirus testing earlier over time is seen for 2019–2022, a finding which should be further monitored in the future in order to enable earlier diagnosis of this severe disease.

Bornavirus-reactive antibodies were higher in serum than in CSF by all tests employed. We here show in addition that especially IFAT antibody levels correlate with the length of disease. Apparently, these antibodies are not protective during human BoDV-1 encephalitis. However, protective neutralizing antibodies were shown to develop against the glycoprotein (G) in rodent infection experiments; antibodies against N and P antigens were non-neutralizing in such rat studies [29]. Antibodies in the IFAT were earlier present in serum than in CSF, findings that were described previously in much smaller cohorts [10, 25]. The difference in sensitivity between serum and CSF in our study here is due to the higher availability of serum samples at an earlier time point than CSF. Seroconversion was seen in a few cases, with antibodies appearing as early as 12 days after symptom onset. However, depending on awareness and the ordering of serology, antibodies were shown in median after 41 days. Thus, the tests are not sensitive enough to detect antibodies well before the onset of encephalitis and are therefore not suitable to screen (still) healthy individuals. A variable time of antibody detection was described before [10, 25, 30]. A late seroconversion in combination with unawareness of the disease delays diagnosis of BoDV-1 encephalitis markedly.

In our study, IgG subclass testing revealed that IgG1 is predominantly produced during BoDV-1 infection in serum. In other forms of viral encephalitis, such as in herpes simplex encephalitis [31] and in patients with Japanese encephalitis [32], IgG1 was also the predominant subclass. IgG1 is in general the most abundant IgG subclass and contains bacterial and viral peptide-specific antibodies with efficient complement activation. Whether testing of IgG1 instead of IgG might improve results in the IFAT warrants further investigation.

Here, we show that patient antibodies in serum and CSF bind to six linear regions on the N antigen and to three linear regions on the P antigen of BoDV-1 which we have mapped on a 3D surface. In order to facilitate handling of bornavirus serology, such peptides might be used in future more high-throughput assays, including enzyme immunoassays (EIAs). However, antibodies reacting with short linear BoDV-1 N and P epitopes were shown to be present also in non-BoDV-1 encephalitis patients in our study, in a 20 year-old investigation [33] and also in a recent epidemiological study [34]. Thus, antibodies against single BoDV-1 antigenetic epitopes may still lack specificity, which has been analyzed more in-depth recently for N, P and X protein epitopes [34]. A combination of several peptide epitopes might possibly help to overcome these obstacles and future studies will therefore have to show how useful such peptides will be when employed in respective assays.

Altogether, the employed tests exhibited a high specificity and high PPVs and NPVs. The IFAT is training-intensive to recognize the typical intranuclear staining pattern but reaches a specificity of 100% when uninfected cells are used in parallel for comparison and respective positive controls are employed. The pattern can be confused with several ANA patterns seen in autoimmune diseases such as nuclear dots, pleomorphic, fine or coarse granular speckles in patients with lupus erythematosus, Sjögren’s syndrome, mixed connective tissue disease, and others [35]. In our test panel with sera encompassing various ANAs, RF and polyclonal B cell stimulation conditions (EBV and CMV infection, malaria), no bornavirus-typical IFAT results were seen. The uninfected cell line employed in parallel proved very useful. Sensitivity of the IFAT in serum and CSF was good, but higher when all samples were tested (including BoDV-1 encephalitis follow-up samples) than only initial samples.

The LB also reaches a specificity of 100% when the respective N and P combination pattern scheme is employed; otherwise unspecificity is seen which is much higher for the N protein than for the P protein. Recently, such unspecifity of antibodies against the N and P antigen were shown to be due to cross-reacting immunoglobulins against human and bacterial proteins [34]. The respective AU height difference of the P antigens of BoDV-1 and the related VSBV-1 was successfully used to serologically discriminate between the two viruses lately [10]. Using the N + P combination pattern proved robust and was not negatively impacted by various autoimmune sera. However, compared to the IFAT, the LB was markedly less sensitive in both serum and CSF, and became later positive during the course of disease in either sample material. No LB positivity was seen without a respective IFAT positivity. LB positivity was more often seen in serum (higher IFAT titers) than CSF (lower IFAT titers) and thus seems to be dependent on the IFAT titer.

Also the qPCR is 100% specific for BoDV-1 and does not react positively in other conditions than BoDV-1 infection. However, as the virus is strongly cell-bound, the sensitivity of the PCR was only 55% in CSF and often low copy numbers (high ct values) were found. Sensitivity further decreased during the course of disease, as BoDV-1 RNA was often no longer detectable in follow-up samples. Of note, the PCR was more often positive in IFAT negative CSF samples than in IFAT positive CSF samples of BoDV-1 encephalitis patients, hinting at a possible role of antibodies (seroconversion) interfering with viral loads in the CSF. However, the individual height of the IFAT titer in CSF did not correlate well with the PCR results, and more samples are needed to analyze this preliminary finding.

## Conclusion

BoDV-1 infections in humans, as reflected by positive serology or viral RNA detection, was only found in encephalitis patients from Borna disease endemic areas. Infection was not associated with other clinical conditions such as neuropsychiatric diseases. We here show that in contrast to high specificity, robustness and clinical/temporal correlation of the diagnostic tests and their respective results, the sensitivity of the assays for human BoDV-1 encephalitis is moderately to markedly lower. Thus, in patients with the clinical suspicion of BoDV-1 encephalitis (severe disease and geographical link to endemic areas), it is strongly advised to test follow-up samples if the initial sample is negative. While these tests nonetheless perform well in clinically ill patients, their sensitivity means that screening of healthy individuals during the incubation time is unlikely to be beneficial. Testing for bornavirus-reactive antibodies employing detection of the IgG1 subclass and using specific peptides may facilitate diagnosis in future assays.

A higher awareness for this nearly uniformly fatal disease is needed among healthcare personnel in order to earlier commence treatment attempts in the future. Recently, a case–control study showed that living in virus endemic areas at the edge of a settlement or amidst fields is a risk factor for BoDV-1 encephalitis with a high odds ratio [36], and the portal of entry for the virus into the human host is possibly the olfactory nerve [20]. With epitope and subclass-specific tests described here, faster diagnosis and more insights into the pathophysiology of BoDV-1 encephalitis may be gained in future studies. Antibody production forms a part of the adaptive host response and upcoming research will address the question whether such antibodies are protective and neutralizing during disease, and if they may be used as therapeutic or post-exposure agents. So far, there is no vaccination for this nearly uniformly fatal disease, and antibody characterizations may form the basis of possible future vaccine development.

## Data Availability

Datasets can be made available on an individual basis; however, ethics regulations apply.
